# *Aspergillus* spp. colonization in exhaled breath condensate of lung cancer patients from Puglia Region of Italy

**DOI:** 10.1186/1471-2466-14-22

**Published:** 2014-02-18

**Authors:** Giovanna E Carpagnano, Donato Lacedonia, Grazia Pia Palladino, Giuseppe Logrieco, Elisabetta Crisetti, Antonia Susca, Antonio Logrieco, Maria P Foschino-Barbaro

**Affiliations:** 1 Institute of Respiratory Disease, Department of Medical and Occupational Sciences, University of Foggia, Foggia, Italy; 2 University of Bari, Bari, Italy; 3 Institute of Sciences of Food Production, Research National Council, Bari, Italy

**Keywords:** Lung cancer, EBC, Fungi, Spores, *Aspergillus*, *Penicillium*

## Abstract

**Background:**

Airways of lung cancer patients are often colonized by fungi. Some of these colonizing fungi, under particular conditions, produce cancerogenic mycotoxins. Given the recent interest in the infective origin of lung cancer, with this preliminary study we aim to give our small contribution to this field of research by analysing the fungal microbiome of the exhaled breath condensate of lung cancer patients from Puglia, a region of Italy.

**Methods:**

We enrolled 43 lung cancer patients and 21 healthy subjects that underwent exhaled breath condensate and bronchial brushing collection. The fungal incidence and nature of sample collected were analysed by using a selected media for Aspergillus species.

**Results:**

For the first time we were able to analyse the fungal microbioma of the exhaled breath condensate. 27.9% of lung cancer patients showed a presence of *Aspergillus niger*, or *A. ochraceus* or *Penicillium* ssp. while none of the healthy subjects did so.

**Conclusion:**

The results confirmed the high percentage of fungal colonization of the airways of lung cancer patients from Puglia, suggesting the need to conduct further analyses in this field in order to evaluate the exact pathogenetic role of these fungi in lung cancer as well as to propose efficient, empirical therapy.

## Background

Lung airways are perpetually exposed to inhaled particulate materials that include pollens, viruses, bacterial and fungal spores [1]. While many of these particles are innocuous, some spores have the potential to germinate and cause invasive lung diseases [1]. A recent theory suggests that infections could also trigger lung cancerogenesis [2]. This hypothesis has been formulated mainly for viruses, although it has been not excluded that also bacteria and fungi could be involved.

If it is possible that the infections could cause lung cancer, it is sure that the lungs of subjects with cancer are particularly susceptible to infection that significantly compromises the patient’s prognosis [2]. The spectrum of pulmonary infections depends on the underlying immunologic deficit or deficits, whereas fungal infections are common if neutropenia persists [3].

Lung cancer is also characterized by airways inflammation, that usually follows cigarette smoking which is likely to play a role in cancer transformation [4,5]. The inflammatory status also further compromises host lungs, promoting tissue invasion and systemic dissemination of the infection [4,5].

The main fungus that could play a role in lung cancer transformation by colonizing the airways is the *Aspergillus*, a mold that forms conidia which, owing to their small size, can bypass mucociliary clearance mechanisms and are inhaled into terminal airways and phagocytosed by alveolar macrophages (AMØs) [6-8]. Toxigenic *Aspergillus* species are common in the Mediterranean environment, colonizing different crops, including maize, grapes and dried fruits [9-11] and are known to be able to produce mycotoxins, such as aflatoxin, ochratoxin A and fumonisins [12], when host plants are stressed by extreme temperature or moisture conditions, poor soil fertility or insect damage.

Despite the potential clinical and therapeutic consequences of the hypotheses of lung cancerogenesis triggered by fungal infection (and particularly by *Aspergillus*) and notwithstanding the prevalent rates of mold colonization in lung cancer patients significantly increased in the last decade [13], no comprehensive studies are available on this field. The few studies present in literature have described airways’ fungal colonization in lung cancer patients or as a one-off report or simply express this as the consequence of the immunodepressive status characteristic of tumours [14-17]. However, several studies are available supporting the viral origin of lung cancer [2,18]. Among these studies, one emerging from our group recently analysed viral colonization in the exhaled breath condensate (EBC) of non-small cell lung cancer (NSCLC) patients, involving a sample from airways that is completely non-invasive and apparently suitable for microbiological studies ( [2], unpublished data). We support the potential of this sample as it contains volatile, soluble and OMIC markers, most of which have already been demonstrated to be useful in the non-invasive diagnosis of several lung diseases, and particularly of lung cancer, which still results in many fatalities, mainly because it is always diagnosed at an advanced stage, as there are no non-invasive screening tools providing a key for its early detection and improving a person’s chances of survival.

With this study we want to give a preliminary contribution to this field of research, giving a view of the incidence and nature of fungal colonisations in lung cancer patients from Puglia and correlating eventual positivity with anthropometric, clinical and oncologic data. Thus, for the first time to our knowledge, we analysed the fungal microbiome in the EBC, comparing results with paired bronchial brushing.

## Methods

### Characteristics of the patients

64 consecutive patients with a suspicion of lung cancer who consented to the study were enrolled at the Unit of Thoracic Surgery, Casa di Cura La Madonnina, Bari and at the Department of Respiratory Disease, Foggia University (Table 1). Written informed consent was obtained from all the subjects upon approval of the study by the Ethics Committees of the University of Foggia.

**Table 1:** Anthropometric, clinical and microbiological data from NSCLC subjects and controls

		**NSCLC**	**Controls**
Number		43	21
Age (Years)		68.4 ± 9.2	64.1 ± 13.1
Sex	Male (%)	26	11
Smoke habit:	Smokers/Ex-Smokers	25	12
	Non smokers	18	9
Histotype:	Squamouscell carcinoma	36	-
	Adenocarcinoma	7	-
Stage:			
I		16	-
II		5	-
III		12	-
IV		10	-
Fugal colonization: Aspergillus niger +	Brushing (%)	11.6%	-
	EBC (%)	11.6%	-
Aspergillus ochraceus	Brushing (%)	6%	-
	EBC (%)	6%	-
Pennicilium ssp	Brushing (%)	9.3%	-
	EBC (%)	9.3%	-

*Abbreviations*: *NSCLC* non small cell lung cancer, *EBC* exhaled breath condensate.

All the patients were enrolled in the study before pathological diagnosis. All of them also underwent standard staging procedures consisting in a physical examination, serum chemistry analysis, brain, chest and abdomen CT scans, and a radionuclide bone scan.

In such cases the definitive diagnosis of malignancy derived from a positive cytohistology of the samples is obtained broncoscopically. Following the histological analysis carried out on specimens, 21 subjects turned out to be negative and were considered controls. In the remaining 43 subjects, the suspicions of lung cancer were confirmed. Squamous cell carcinoma was diagnosed in 36 (83.7%) subjects, whereas 7 (16.2%) subjects were found to be affected by adenocarcinoma. Overall the NSCLC patients 16 (37.2%) were classified as stage I, 5 (11.6%) as stage II, 12 (27.9%) as stage III and 10 (23.2%) as stage IV.

All the subjects underwent EBC and bronchial brushing collection (the latter was carried out during bronchoscopy). All subjects underwent a fungal investigation in these samples. Type and frequency of these colonisations were analyzed.

Information on their smoking habit was acquired at the time of diagnosis.

Twenty-five of the NSCLC patients were current/ex-smokers (58.1%, 43.3 ± 25.2 pack/year), of whom 8 ex-smokers (32%) had quitted smoking 9.65 ± 8.5 years previously, whereas 18 were non-smokers. A detailed history relating to their family history of lung cancer or any other cancer was collected in a pre-tested pro-form.

### Bronchial brushing collection and processing

Bronchial brushing specimens were collected in an ice-cold phosphate-buffered saline solution during fiberoptic bronchoscopy. The sample was transferred on freshly prepared Dichloran Rose-Bengal Chloramphenicol Agar (DRBC, Oxoid) medium. The plates were incubated at 25°C for 7 days and examined daily.

### EBC collection and processing

1 mL of EBC in one setting from each patient at the time of diagnosis.

The EBC was collected by using a condenser (EcoScreen Jaeger, Wurzburg, Germany). The condensate was transferred on freshly prepared Dichloran Rose-Bengal Chloramphenicol Agar (DRBC, Oxoid) medium. The plates were incubated at 25°C for 7 days and examined daily.

### Fungal culture and analysis

*Aspergillus* colonies were subcultured for subsequent identification to species level. Species names were determined by following the taxonomic keys of Klich [18]. Culture were incubated for 7 days on CYA (Czapek Yeast Extract Agar) at 25°C and 37°C, CZ (CzapekDox Agar) and MEA (Malt Extract Agar) at 25°C to enable morphological features exploited as diagnostic characters. *Penicillium* colonies were identified only at genus level.

### Statistical analysis

In order to assess the association between categorical variables such as sex, smoking habit or fungi positivity in the brushing or in the EBC and positivity to lung cancer or between histological subtypes or TNM stage and mold positivity, the chi square test (or Fisher exact’s test, when necessary) were calculated. T-student was used for independent samples in order to assess the differences in continuous variables (age, number of pack/years smoked) between lung cancer patients and controls.

A p value of < 0.05 was considered statistically significant.

## Results

Lung cancer cases and healthy subjects enrolled in the present study numbered 43 and 21, respectively. Demographic and clinical data of study subjects are summarized in Table 1.

We were able to detect a fungal colonization by *Aspergillus niger*, *Aspergillus ochraceus* and *Penicillium ssp* in the EBC.

12 (27.9%) of NSCLC patients, and 0 (0%) controls turned out to be fungi colonized in the EBC: 5 by *Aspergillus niger* (11.6%), 3 by *Aspergillus ochraceus* (6%), 4 by *Penicillum spp*. (9.3%).

Fungi positivity in the EBC was always confirmed in paired bronchial brushing. The EBC was found to have a similar sensibility compared to the bronchial brushing for fungi detection.

When analyzing lung cancer patients no difference was found according to sex, age, histotype, stage, smoking habit, pack years, time since quitting smoking in subjects with fungal colonization (p > 0.05).

## Discussion

This was a preliminary study that aimed to analyse the incidence and nature of fungal colonization in lung cancer patients from Region Puglia of Italy. For the first time to our knowledge, we tested fungal microbioma of exhaled breath condensate and paired bronchial brushing of patients with diagnosed lung cancer and of healthy subjects and in 27.9% of lung cancer patients we detected the presence of mold (A*spergillus niger, A. ochraceus and Penicillium ssp*), but not in any of the healthy subjects. The fungal positivity in airways didn’t correlate with the oncologic data.

It has been suggested that the fungal colonization could contribute to or trigger - as do other virus infections (HPV, CMV etc) - pathophysiologic processes associated with lung cancer [19]. However, while there is evidence of the involvement of specific fungal species in asthma, chronic obstructive pulmonary disease (COPD) [20,21] and cystic fibrosis (CF) [21-24], little is known of the airway fungal microbiota in the pathogenesis of lung cancer [19].

Given the nascent of airway microbiome research in lung cancer and some recent efforts to advance these areas, this article has analysed fungal microbiota in the EBC and bronchial aspiration of lung cancer patients from Region Puglia of Italy [25-29]. There are only a few studies that have investigated fungal colonization in the airways of lung cancer patients [14] using bronchial aspirates [14], bronchoalveolar lavage [30], sputum [15] or lung tissue [17]. All these studies have described fungal positivity as a one-off report or as the result of the immunodepression.

Bearing in mind that identifying other risk factors (more than the smoking habit) could have socio-economic consequences for lung cancer, offering a useful tool for prevention programs that are still in a state of bankruptcy, we have designed this preliminary study with the aim of analysing the possible fungal involvement in lung cancerogenesis.

In consideration of the usefulness of using non-invasive methods to analyse the airways of these patients feeling the brunt of several diagnostic tests and often of surgery, we tested the fungal microbiome for the first time in the EBC. Our group previously demonstrated the possibility to analyse viral colonization in this sample of patients affected by lung cancer and supported the recent theory on a possible infective aetiology of lung tumours. In this study we sought to direct our efforts to the identification of the new cancerogenic microrganisms in the EBC, toward which the screening programs could be directed, by testing fungal microbiome.

We found a positivity for A*spergillus niger and A. ochraceus in samples from lung cancer patients, which are species of fungi that may release cancerogenic mycotoxins such as* ocratoxin A e fumonisins. These findings led us to suggest that also these microorganisms and their toxic secondary metabolites may play a role in lung cancer development, although further studies are needed to support this hypothesis.

Furthermore, we observed that fungal positivity in this sample was always the same as had been observed in paired bronchial brushing. The overlap of molds in EBC and bronchial brushing proved that the EBC contains identical information on airway colonisation as previously demonstrated for HPV [2], CMV, EBV (unpublished data) and somatic DNA alterations specific to lung cancer [31], but also further supported the high sensibility and specificity of this sample, confirming its potential usefulness in oncological clinical practice.

We were unable to find any correlation between fungi positivity and sex, age, histotype, stage, smoking habit of subjects studied that would help us in the definition of the pathogenetic role of fungi identified in lung cancer patients. However, the number of patients enrolled in this study was low and justified our results, which we intend to verify on a larger population.

Another recognized limit of the study was not to have tested the presence of fungi in paired plasma, something that might help us to better interpret the origin of fungi in the airways of patients with lung cancer and also its significance.

Further very important analyses should be addressed also to analyze fungal genotypes isolated, in order to asses their ability to produce toxins, and above all, to evaluate the effective presence in human fluids or in lung tumor tissue of mycotoxins potentially produced by *Aspergillus* species isolated.

This was just a preliminary study that will be followed by a genomic and epigenomic characterization and mycotoxin analysis, which we hope will help us in defining the association between this mold, lung cancerogenesis and the pathogenetic mechanisms involved.

We further are planning to better study whether the fungi colonization is the cause or consequence of lung cancer investigating the immunologic status of the fungi-positive patients through the analysis of the density of infiltrating inflammatory cells in surgical tissue samples and the dosage of the plasmatic immunoglobin concentration.

## Conclusions

In conclusion, it can be stated that we have demonstrated that the exhaled breath condensate is a suitable sample, even for the fungal colonization of airways. Furthemore, we found that 28% of lung cancer patients from Region Puglia of Italy showed *Apergillus niger*, *Aspergillus ochraceus* and *penicillium* spp in their airways, opening up future perspectives in this field.

With this study we couldn’t verify whether fungal colonization is the cause or consequence of lung cancer but we can offer a broader view on the infective status of airways of lung cancer patients. In consideration of the potential of our results, we support any future studies in this emerging field that could open up the possibility of microbiota manipulation as a novel therapeutic strategy for screening, treatment or management of lung cancer.

## Abbreviations

EBC: Exhaled breath condensate.

## Competing interest

There aren’t financial disclosures from any of the authors.

## Authors’ contributions

GEC designed the present study and drafted the manuscript. DL enrolled patients. GPP carried out bronchial brushing analyses. GL enrolled patients. EC enrolled patients. AS participated in Fungal culture and analysis. AL participated in Fungal analysis and designed the present study. MPFB reviewed the manuscript. All authors read and approved the final manuscript.

## Pre-publication history

The pre-publication history for this paper can be accessed here:

http://www.biomedcentral.com/1471-2466/14/22/prepub
